# Bioactivity of Samsum ant (*Pachycondyla sennaarensis*) venom against lipopolysaccharides through antioxidant and upregulation of Akt1 signaling in rats

**DOI:** 10.1186/1476-511X-11-93

**Published:** 2012-07-23

**Authors:** Hossam Ebaid, Mohamed Al-Khalifa, Ahmed M Isa, Saad Gadoa

**Affiliations:** 1 Department of Zoology, College of Science, King Saud University, P.O.Box 2455, Riyadh, 11451, Saudi Arabia; 2 Department of OB GYNE, College of Medicine, King Saud University, P.O.Box 7805, Riyadh, 11472, Saudi Arabia; 3 Permanent address: Department of Zoology, Faculty of ScienceEl-Minia University, Minya, Egypt

**Keywords:** AKT1, Samsum ant venom, Oxidative stress, Anti-inflammatory, Lipopolysaccharides

## Abstract

**Background:**

This study aimed at investigating the oxidative stress ameliorating effect, lipids profile restoration, and the anti-inflammatory effect of Samsum Ant Venom (SAV) in induced endotoxemic male rats, injected with bacterial lipopolysaccharides (LPS).

**Results:**

Results revealed that LPS significantly increased the oxidative stress indications in LPS-injected rats. A significant increase of both malondialdehyde (MDA), and advanced oxidative protein products (AOPP), as well as a significant suppression of glutathione were all detected. Treatment with 100 μg/kg dose of SAV significantly restored the oxidative stress normal indications and increased the total glutathione levels. Treatment of the LPS-rats with 100 μg/kg dose of SAV showed a clear anti-inflammatory function; as the histological architecture of the hepatic tissue was partially recovered, along with a valuable decrease in the leukocytes infiltrated the hepatic tissues. Treatment of some rat groups with 600 μg/kg dose of SAV after LPS injection induced a severe endotoxemia that resulted in very high mortality rates. SAV versus the effects of LPS on AKT1, Fas, TNF-α and IFN-γ mRNA expression. SAV was found to significantly lower Fas gene expression comparing to the LPS group and restore the level of IFN-γ mRNA expression to that of the control group.

**Conclusion:**

In conclusion, SAV, at the dose of 100 μg/kg body weight, maintained and restored the oxidative stability, the anti-inflammatory, and the hypolipidemic bioactivity in rats after induced disruption of these parameters by LPS injection. This improvement by SAV was mediated by upregulation of AKT1.

## Background

Most ant species are partially herbivorous, feeding on pollen, extrafloral nectar and food bodies, or are considered “cryptic herbivores” when they attend sap-sucking hemipterans for their honeydew [1]. Secretions used both defensively and offensively are particularly potent [2]. Toxicity tests which have been conducted on five Crematogaster species have shown a repellent activity of the venoms of *C. scutellaris*[3], *C. sp. prox. Abstinens*[4], *C. distans*[5]**,***C. brevispinosa rochai*[6]**,** and other ants.

In Australia, allergy to *Myrmecia pilosula* venom is common, and Venom immunotherapy is a highly effective treatment [7]. Collingwood reported Samsum ant (*Pachycondyla sennaarensis*) for the first time in Saudi Arabia in 1985 [8]. Since that time, there have been further reports on their distribution [9].

Alsharani et al.. [10] presented series of cases of allergic reactions following insect stings, caused by the black samsum ant (*Pachycondyla sennaarensis*). Reactions ranged from mild allergic reactions to severe anaphylactic shock. They indicated that ant stings were a significant public health hazard in Saudi Arabia, and reported that physicians in the Middle East and Asia need to be aware of ant stings as a cause of severe allergic reactions.

Sting of order Hymenoptera can cause reactions ranging from mild local reaction with painful erythromatous swelling to severe life-threading anaphylaxis, as reported by Potier et al. [11]**,** while Al-Shahwan et al. [9] confirmed the anaphylaxis effect of *P. sennaarensis* in Saudi Arabia.

Some reports proved that ant venom possesses many pharmacological effects as reducing inflammation, relieving pain, inhibiting tumor growth, proving the immunological function, liver protection, and hepatitis treatment [12]**.** It was confirmed that the Samsum ants, *Polyrhachis lamellidens* venom exert a potent analgesic and anti-inflammatory action on mice models [13]. SAV has anti-inflammatory effects against xylene-induced dermal inflammation and swelling in the mice ear-skin test [14]. In addition, the anti-tumor effect of SAV was examined on the growth arrest/apoptosis induction of MCF-7 cells as well as its ability to alter the insulin-like growth factor-1 (IGF-1) mediated breast cancer cell proliferation and to detect the underlying mechanisms [15]. Their data revealed a unique anti-tumor effect of the venom, which can be a new approach that enhances immunogenicity, suppresses cell proliferation, and increases apoptosis of cancer cells.

The potential role of SAV in diseases treatment in vivo needs further intensive investigation. In the present work, we investigated the anti-oxidant, the anti-inflammatory and the hypolipidemic bioactivities of this promising ant venom, after induction of acute bacterial endotoximic septic shock in rat models.

## Methods

### Experimental Animals

Sixty adult rats weighing 80–100 g were obtained from the faculty of pharmacy, king Saud University, Saudi Arabia. The animals were then housed in stainless steel wire cages (5 animals/ cage), under pathogen-free conditions. All animal procedures were in accordance with the standards set forth in the guidelines for the care and use of experimental animals by the Committee for Purpose of Supervision of Experiments on Animals (CPCSEA), and the National Institutes of Health (NIH) protocol. The Animal Ethics Committee of the Zoology Department, College of Science, King Saud University, approved the study protocol. Animals were maintained at 18–22 °C on a 12:12 hr light/dark cycle, and were provided with food and water ad libitum.

### Dissection of the venom gland, sepsis model and treatment with SAV

The sting apparatus was removed by grabbing the last segment of the abdomen and detaching it with the sting apparatus. The venom gland was pinched out and placed in a small tube [16]. Glands were homogenized, and then centrifuged at 1000 rpm for 2 min. and the supernatant was collected.

Rats were divided into six groups, ten rats each. The first was the untreated negative control group. Rats of the second (LPS group), third (LPS + 100 SAV), and fourth (LPS + 600 SAV) groups had intra-peritoneal injection with the lipopolysaccharide (LPS) at a single dose of 2.5 mg/kg. The third and fourth groups were further treated with SAV at doses of 100 and 600 μg/kg, respectively. The fifth (100-SAV) and the sixth (600-SAV) were the positive control groups that were treated, also daily for one week, with only SAV, at doses of 100 and 600 μg/kg, respectively. A dose of 10 μl/mouse was previously applied by Dkhil et al. [14]. In this study, we applied several doses from the freshly prepared crude venom and doses which exhibit significant changes were chosen, diluted with 250 μl PBS and injected via the intraperitoneal route.

### Blood samples, plasma and liver

Animals were anesthetized with pentobarbital (60 mg/kg body weight) and samples (blood and liver tissues) were obtained at the end of the experiment. A part of the liver was stored at −80 °C for PCR analysis. Whole blood was drawn from the abdominal aorta. Half of the obtained blood was used to evaluate the complete blood picture and the differential count of the white blood cells.

### Glutathione activity associated with endotoxemia and SAV bioactivity

A glutathione (GSH) assay was carried out on the tissue samples according to Clark *et al*. [17]. Briefly, liver of the tested animals were removed and gently rinsed in physiological saline (0.9% NaCl). Fresh weights were recorded and the organs were frozen at −20 °C until used. A 10% (w/v) homogenate of each frozen tissue was prepared and the supernatant was used for oxidative stress evaluations. The resulting supernatant was boiled to deactivate and precipitate other proteins, but this did not alter GSH levels. GSH concentrations were then measured by adding 100 μl of boiled supernatant to 400 μl PBS. GSH concentrations were then determined by measuring the absorbance (OD) of the reaction after 1 min at 340 nm using a UV Visible Spectrometer (Ultrospec 2000, Pharmacia Biotech). GSH standards were measured concurrently to obtain a standard curve that was used to calculate GSH concentrations in samples. Results were expressed as μg GSH/g tissue. Statistical comparisons of GSH activities between controls and treatments in each case were performed using the Minitab statistical program as detailed below.

### Determination of lipid peroxidation

Endogenous lipid peroxidation in tissues homogenates was estimated spectrophotometrically following the method described by Okhawa *et al.*[18] and expressed in nano-moles of malondialdehyde (MDA) per milliliter homogenate (nmole/ml). Tissues were homogenized as described above for the glutathione method. An aliquot of 0.5 ml of the resulting supernatant was shaken with 2.5 ml of 20% trichloroacetic acid (TCA). To the resulting mixture, 1 ml of 0.67% thiobarbituric acid (TBA) was added, shaken, and warmed for 30 min in a boiling water bath, and followed immediately by rapid cooling in ice for 5 min. After cooling, 4 ml of n-butyl-alcohol was added and the sample was shaken well. The resulting mixture was then centrifuged at 16,000 × g for 5 min. The resultant n-butyl-alcohol layer was transferred to a separate tube and MDA content was determined spectrophotometrically at 535 nm using a UV Visible Spectrometer (Ultrospec 2000, Pharmacia Biotech).

### Determination of advanced oxidation protein products (AOPP)

AOPP levels were determined according to the method of Kayali et al. [19]. Briefly, 0.4 ml of pancreatic supernatant was treated with 0.8 ml phosphate buffer (0.1 M; pH 7.4). After 2 min, 0.1 ml 1.16 M potassium iodide (KI) was added to the tube followed by 0.2 ml of acetic acid. The absorbance of the reaction mixture was immediately recorded at 340 nm. The concentration of AOPP for each sample was calculated using the extinction coefficient of 261 cm_1 mM_1 and the results were expressed as nmol/mg protein.

### Plasma lipids profile and liver enzymes

Lipids profiles were determined colorimetrically with BioMerieux kits and a standard assay method. Cholesterol levels were evaluated using the cholesterol esterase method [20]. Liver enzymes (ALP, GOT, GPT, GGT) were measured using commercial kits (Labtest Diagnostica, Brazil) according to the manufacturer’s instructions.

### Histological sections

Liver parts were collected from the sacrificed control and different treated rats groups. Tissues were fixed in Bouin’s fixative, processed into paraffin, and 4 micrometer thick sections were prepared. Sections were stained with Haematoxylin and Eosin (H&E) for general histological architecture. In each group, many sections from different rats were investigated and the clear and common changes were photographed. Only sections from two rats of LPS + 600 SAV group were considered because the rest did not survive the experiment to the end.

### RNA extraction and cDNA synthesis

Total RNA from liver tissues homogenate was isolated using TRIzol reagent (Invitrogen®). The isolation was performed according to the manufacturer's instructions and quantified by measuring the absorbance at 260 nm; the RNA quality was determined by measuring the 260/ 280 ratio. The cDNA synthesis was performed using the High-Capacity cDNA reverse transcription kit (Applied Biosystems®), according to the manufacturer's instructions. 1.5 μg of total RNA from each sample was added to a mixture of 2.0 μ1 of 10x reverse transcriptase buffer, 0.8 μl of 25x dNTP mix (l00 mM), 2.0 μl of 10x reverse transcriptase random primers, 1.0 μl of MultiScribe reverse transcriptase, and 3.2 μl of nuclease-free water. The final reaction mixture was kept at 25 °C for 10 min, heated to 37 °C for 120 min, heated for 85 °C for 5 s, and then cooled to 4 °C.

### Quantification of mRNA expression by real-time polymerase chain reaction (RT-PCR)

Quantitative analysis of mRNA expression of target genes was performed by RT-PCR. cDNA from the above preparation was subjected to PCR amplification using 96-well optical reaction plates in the ABI Prism 7500 System (Applied Biosystems®). The 25-μl reaction mixture contained 0.1 μl of 10 μM forward primer and 0.1 μl of 10 μM reverse primer (40 μM final concentration of each primer), 12.5 μ1 of SYBR Green Universal Mastermix, 11.05 μ1 of nuclease-free water, and 1.25 μ1 of cDNA sample. The primers used in the current study were chosen from pubmed.com. The RT-PCR data was analyzed using the relative gene expression method, as described in Applied Biosystems® User Bulletin No. 2. The data are presented as the fold change in gene expression normalized to the endogenous reference gene and relative to a calibrator.

### Statistical analysis

Statistical analysis was done using MINITAB software (MINITAB, State College, PA, Version 13.1, 2002). Treatment of the fourth group with SAV at dose of 600 μg/kg after LPS resulted in very high mortality rates. Thus, this group was excluded from all statistical analysis and presented data. Data from experiments were first tested for normality using Anderson Darling test, and for variances homogeneity prior to any further statistical analysis. Data were normally distributed, and variances were homogeneous, thus, One-way ANOVA was used to determine overall effects of treatments followed by individual comparison using Tukey’s Pairwise comparison.

## Results

### Mortality rate in different animal groups

Induction of acute endotoxemia in rats with LPS (2.5 mg/kg) synergistically with 600 μg/kg dose of SAV (LPS + 600 SAV) caused 80% mortality (Table 1). On contrary, no morphological changes were observed in the rest of all groups; only normal life activity was observed with them all.

**Table 1:** Mortality in different studied groups resulted from the LPS challenge and the effect of SAV

	**Control**	**LPS**	**LPS + 100**	**LPS + 600**	**100**	**600**
			**SAV**	**SAV**	**SAV**	**SAV**
Number	10	10	10	10	10	10
Mortality	0	1	0	8	0	0
Mortality%	0	10	0	80	0	0

### Effects of SAV versus LPS on oxidative stress

**Figure 1:**
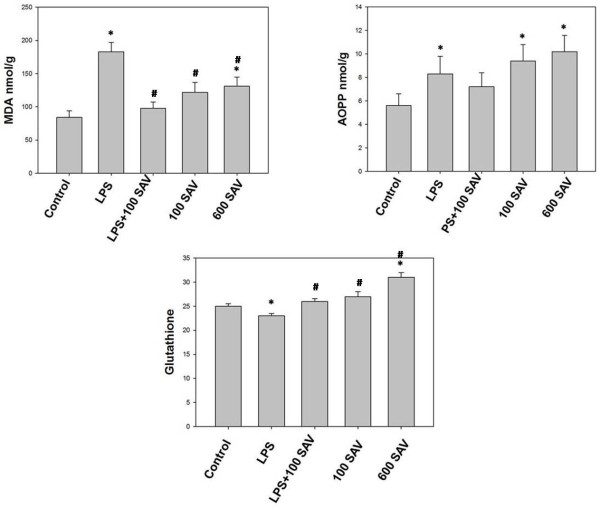
**Changes of lipid peroxidation (Malondialdehyde, MDA), Advanced oxidation protein products (AOPP) and total glutathione levels.** MDA and AOPP levels were found to be significantly higher in the LPS–treated and 600 μg/kg SAV–treated groups than in control group. SAV was found to significantly restore the oxidative stress in LPS + 100 SAV group. Glutathione level was significantly suppressed in LPS-treated group followed by a restoration effect after treatment with SAV in 100 μg/kg SAV group. Values are expressed as the means ± standard errors. * shows statistically significant differences at P < 0.05 from the control and # shows statistically significant differences at P < 0.05 between any group comparing to the LPS group.

Because free radicals are the culprits in the pathophysiological processes, we monitor oxidative stress in different treatments of this study. We found a significant increase in the endotoxemia-induced oxidative stress in terms of the levels of MDA and AOPP in two groups, the LPS-only-treated group, and the 600 μg/kg SAV-only-treated group. Levels of MDA were 2-fold and 1.5-fold in the LPS-only and 600 μg/kg SAV-only groups, respectively (Figure 1). AOPP levels were 1.5-fold and approximately 2-fold in the LPS-only and the 600 μg/kg SAV-only groups, respectively, in relation to the control group. On the other hand, SAV in the LPS + 100 SAV group significantly restored the oxidative stress (in terms of MDA and AOPP levels) to the normal control levels. The anti-oxidant glutathione levels were significantly suppressed in LPS-only-treated group followed by a significant restoration of its level in the SAV 100 μg/kg- treated group (Figure 1).

### **Effects of SAV versus LPS on***mRNA* expression of AKT1, Fas, IFN-γ and TNF-α

**Figure 2:**
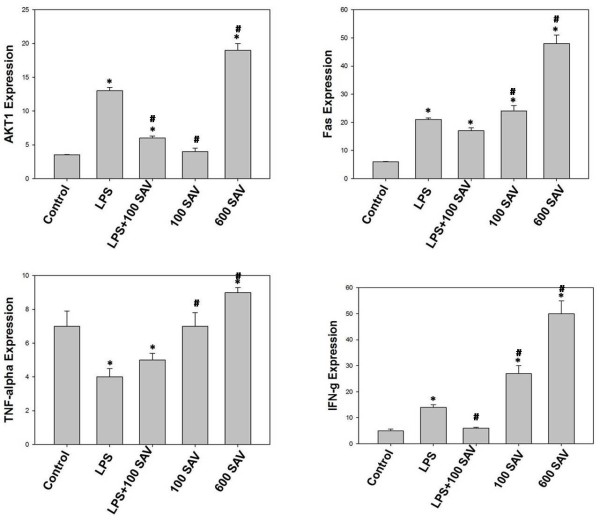
**Effects of SAV versus LPS on mRNA expression of AKT1, Fas, TNF-a and IFN-g.** Values are expressed as the means ± standard errors. * shows statistically significant differences at P < 0.05 from the control and # shows statistically significant differences at P < 0.05 between any group comparing to the LPS group.

Control of the redox environment of the cell provides for additional regulation in relation to critical cellular signal transduction pathways [21]. Thus, we expected that AKT1, Fas, IFN-γ and TNF-α mRNA expression will be regulated by oxidative stress. LPS was found to significantly elevate each of AKT1, Fas and IFN-γ mRNA expression comparing to the control group. However, LPS significantly suppressed gene expression of TNF-α. Interestingly SAV versus the effects of LPS on these genes. SAV was found to significantly lower both AKT1 and Fas mRNA expression in LPS + 100 SAV group comparing to the LPS-only group, although both genes were significantly higher expressed than those of the control group. In addition, SAV was found to partially and completely restore the levels of TNF-α and IFN-γ mRNA expression in LPS + 100 SAV group to those of the control group (Figure 2). 100 SAV-rats revealed a normal levels of AKT1 and TNF-α with significantly higher mRNA expression of Fas and IFN-γ. 600 SAV-rats showed significant upregulation of all studied mRNA expression. Remarkably, IFN-γ mRNA expression in 100 SAV-only group was significantly elevated indicating that SAV increased the polyfunctional T cells (IFN- γ- and IL-2-producing cells that exhibit a high proliferation capacity).

### Effects of LPS and SAV on blood picture

Results of blood parameters revealed that bacterial LPS-induced acute septic shock resulted in a significant decrease in the count of RBC, blood platelets and lymphocytes (Table 2). It also caused a significant increase of the total WBC and neutrophil counts. On the other hand, results showed clearly that the SAV-reactivity in LPS + 100 SAV group was directed against the inflammatory bio-reactivity of the endotoxic LPS since, a decrease in the total and differential leukocytic count, in particular lymphocyte count, was significantly observed as represented in Table 2.

**Table 2:** Changes in blood parameters caused by LPS challenge and SAV treatment

	**Control**	**LPS**	**LPS + 100**	**100 SAV**	**600 SAV**
			**SAV**		
RBC X10^6^ /mm^3^	6 ± 0.3	5 ± 0.6*	4.2 ± 0.9*	5.2 ± 0.9	5.8 ± 0.4
PCV%	33 ± 2.5	38 ± 3.7*	34 ± 3.3	39 ± 2.9*	41 ± 3.7*
Hb mg/100 mg	13.3 ± 0.7	12.7 ± 0.6	11.2 ± 0.5	12.9 ± 0.5	13.7 ± 0.3
Platelet x10^5^/mm^3^	9 ± 1.2	6 ± 0.7*	12 ± 2.9	8 ± 1.01*	6.7 ± 0.9*
WBC x10^3^/mm^3^	9.7 ± 1.08	12 ± 1.1*	6.8 ± 0.5*	10 ± 0.98	11.8 ± 1.2*
Neut. x10^3^/mm^3^	1.5 ± 0.41	3.5 ± 0.8*	2.2 ± 0.23*	2.7 ± 0.5*	3.2 ± 0.5*
Lymph. x10^3^/mm^3^	8 ± 0.78	6.4 ± 0.8*	3.8 ± 0.2*	5 ± 0.51*	6 ± 0.7*

Values are expressed as the means ± standard errors. * shows statistically significant differences at P < 0.05 from the control. RBC: Red blood cells; PCV: Packed cell volume; Hb: Hemoglobin; WBC: White blood cell; Neut.: Neutrophil; Lymph.: Lymphocytes.

### Hypolipidemic effect of SAV

**Figure 3:**
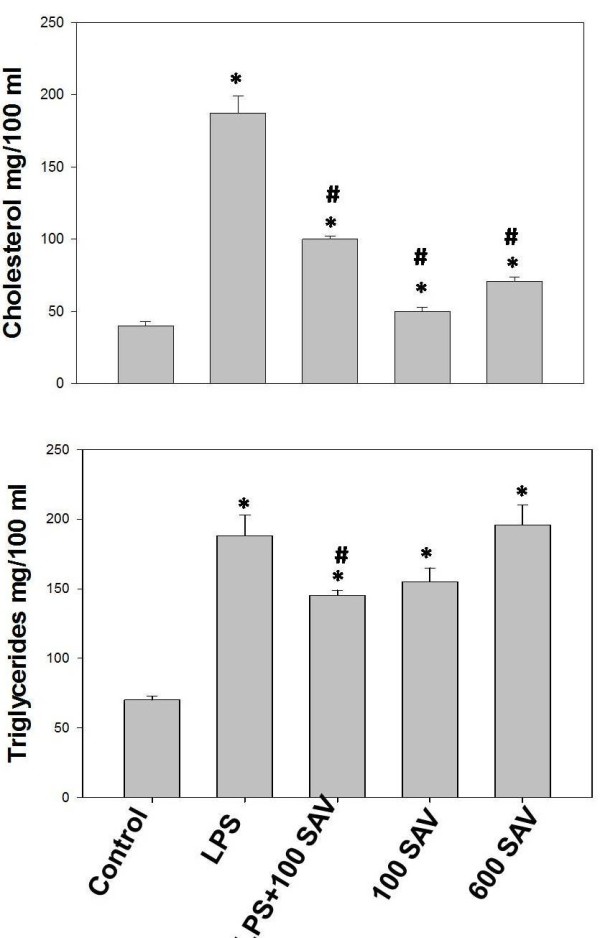
**Triglycerides and total cholesterol changes resulted by the LPS challenge and treatment of SAV in serum.** Values are expressed as the means ± standard errors. * shows statistically significant differences at P < 0.05 from the control and # shows statistically significant differences at P < 0.05 between any group comparing to the LPS group.

Blood lipid level is a significant indicator on the ability of liver to uptake different lipid derevatives. LPS, in the LPS-only-treated group, was found to significantly (P < 0.05) elevate the level of cholesterol in plasma to 4-fold that of the control group. SAV, in the LPS + 100 SAV group, significantly restored 50% of the control cholesterol levels. In addition, SAV remarkably restored the triglyceride levels to the control levels, after they were significantly elevated by LPS in the LPS-only-treated group. On the other hand, in 600 SAV group, SAV significantly (P < 0.05) increased the level of both cholesterol and triglycerides with 1.5-fold and 4-fold, respectively, compared to that of the control group (Figure 3).

### Hepatic histopathological and physiological investigations

**Figure 4:**
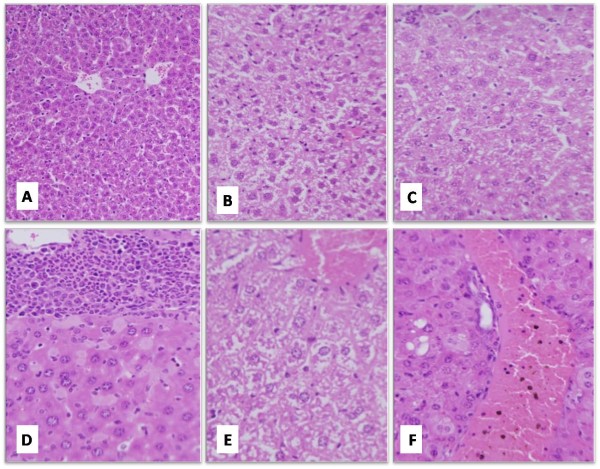
**Histological features of a representative liver section from control animal group (A; H&E X200).** A representative section from LPS-treated group showing the increased vacuolation (**B**; H&E X400). SAV showed a remarkable improvement in the histological architecture of LPS + 100 SAV group (**C**; H&E X400). Liver sections from LPS + 600 SAV group showing an inflammatory hyperplasia with a fragmentation in the chromatin materials **(D**; H&E X400). Vacoulations with hemorrhage and dilation in the central veins were detected in liver sections from 600 SAV group (**E**; H&E X1000). Fragmentation of the chromatin and hemorrhage with hemosiderin granules and dilation in the central veins were noted in the 600 SAV group (**F**; H&E X400).

Examination of the liver sections from LPS-treated groups showed intense hepatic tissue damage. Liver lost its characteristic architecture compared to the control group since inflammatory cellular infiltration was abundant around the central vein (Figure 4). In addition, the cytoplasm of the hepatocytes was characterized by having coarse, pink, darkly stained granules and few vacuoles. SAV treatment, in the LPS + 100 SAV group, significantly restored the hepatic tissue features, where the boundaries between hepatocytes were clear with non-vacuolated cytoplasm and normally appeared nuclei. In addition, SAV reduced the number of infiltrated inflammatory cells and remarkably increased the Kuppfer,s cells in the hepatic tissues. On the other hand, sections taken from the only two rats of LPS + 600 SAV-group (mortality in this group was 80 were mainly characterized with hyperplasia and fragmentation of the chromatin material of hepatocytes. Furthermore, the high dose of SAV at 600 μg/kg body weight (the positive control, group number six) caused vacuole-formation in the hepatocytes cytoplasm and a hemorrhage with brown hemosiderin granules (Figure 4).

**Figure 5:**
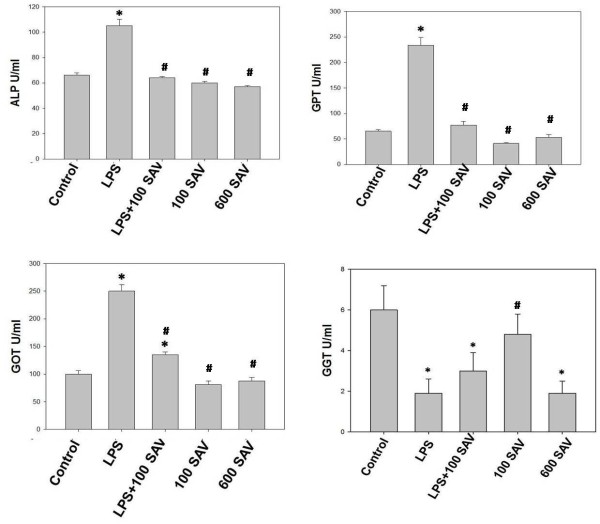
**Liver enzymes (ALP, GOT, GPT, GGT) resulted by the LPS challenge and treatment of SAV in serum Liver enzymes (ALP, GOT, GPT, GGT) resulted by the LPS challenge and treatment of SAV in serum.** Enzymes were found to significantly record high levels in the LPS-treated group. SAV restores the values of ALP, GOT, GPT in LPS + 100 SAV group. SAV slightly improved GGT values in LPS + 100 SAV group. Values are expressed as the means ± standard errors. * shows statistically significant differences at P < 0.05 from the control, and # statistically significant differences at P < 0.05 between any group comparing to the LPS group.

Liver enzymes (ALP, GOT, GPT), in the LPS-only-treated group, recorded highly significant levels, 1.5-fold, 2.5-fold, and 4-fold the control values, respectively. SAV supplementation (LPS + 100 SAV group) significantly restored these values to their control counterparts (Figure 5). Surprisingly, SAV supplementation, when taken alone at either dose, did not record any significant changes of the liver enzyme levels. In contrast, GGT, as a marker of liver fibrosis and cirrhosis, was significantly affected by most treatments in all studied groups. Unlike the other liver enzymes (ALP, GOT, GPT), LPS significantly suppressed the GGT enzyme which appeared in a very low value compared to the control. However, the SAV supplementation, in LPS + 100 SAV group, did partially restore the GGT value to the normal level (Figure 5).

## Discussion

This study investigated the oxidative stress, blood lipid-profile and the anti-inflammatory effects of SAV in male rats. The low dose (100 μg/kg body weight), but not the high dose (600 μg/kg body weight), of SAV significantly ameliorated the damage effects of LPS via the anti-oxidant and the anti-inflammatory bioactivities. Therapeutic efficacy of the ant *Polyrhachis lamellidens* venom was confirmed. It was reported to exert potent analgesic and anti-inflammatory actions in the treatment of various inflammatory disorders [13]. In addition, many studies proved that different ant venoms possess many pharmacological effects as reducing inflammation, relieving pain, inhibiting tumor growth, proving the immunological function, and for liver protection and treatment against hepatitis [12]. Many active substances such as citral, ATP, histamine, growth hormone, testosterone, and superoxide dismutase characterize the ants’ venom [13]. Ants share some common proteins in venoms, but each group appears to have a number of poss0069bly unique components. Further proteomic studies should expand and clarify our knowledge of these fascinating animals [22]. Phenol-2, 4-bis (1,1 dimethylethyl) and trimethyl pyrazine are the main volatile components of the venom gland of Samsum ants [16].

Here, we found that the concentration of MAD and AOPP, which are a significant index of oxidative stress, were elevated in LPS-only-treated rats [23]. The increase of the free radicals resulted in high levels of MAD and AOPP as was proved in our study [24]. This result was confirmed with the intense tissue damage in the liver sections, since the liver lost its characteristic architecture and inflammatory cellular infiltration was abundant around the central vein which in turn, lead to a liver dysfunction. These results are in accordance with those previously obtained [25]. It was clear that liver enzymes were significantly high in LPS-only-treated group. The antioxidant glutathione, which could scavenge these free radicals, was very low in LPS-only-treated group. Therefore, LPS induced an oxidative imbalance, as previously found [26]. It was found that LPS increased MDA, and nitric oxide and thus, organ damage [27]. Therefore, complications recorded in this study were caused by the actions of free radicals which damaged cellular components such as lipids, proteins and DNA [28]. Thus, oxidative stress is a potential cause of the different tissue damage and the impairment of different physiological and biochemical activities in the body.

Our data revealed that SAV improved endotoxic complications because it significantly decreased the elevated levels of MAD and AOPP. Oxidative stability was evident with the markedly elevated glutathione level after treatment with the low dose of SAV. In accordance, a hepatic tissue improvement along with normal liver functions was observed in this group. It was confirmed that the SAV exert a potent analgesic [13], anti-inflammatory [14] and anti-tumor effects [15]. Solenopsin A, a venom alkaloid from the fire ant *Solenopsis invicta*, exhibits antimicrobial activity against gram-positive bacteria [29]. The antimicrobial, insecticidal, and hemolytic properties of peptides isolated from the venom of the predatory ant *Pachycondyla goeldii*, a member of the subfamily Ponerinae, were proved [30].

ROS is needed to upregulate NF-κB which in turn is required for the induction of pro-inflammatory cytokines, such as IL-1ß, TNF-α and IL-6 [31]. Although we did not address NF-κB level in the present study, oxidative stability induced by SAV may mediate the downregulation of NF-κB, leading to the suppression of the inflammatory cascade and the low level of TNF-α as was found in the current study. TNF-α is a key mediator of immune and inflammatory responses, and it controls the expression of the inflammatory gene network. Overproduction of TNF-α contributes significantly to the pathological complications observed in many inflammatory diseases. For example, pro-inflammatory cytokines can increase the risk of schizophrenia [32]. Thus, supplementation with SAV has broad anti-inflammatory effects and attenuates allergic inflammation in LPS-treated rats in the current study. Similarly, the anti-inflammatory thymoquinone inhibited the transport of NF-κB from the cytosol to the nucleus [33]. Overproduction of pro-inflammatory cytokines is likely to contribute to manifestation of the systemic inflammatory response, and hence the development of organ failure [34]. This clearly explains the hepatic tissue damage and dysfunction in the LPS-treated group in this study. On the other hand, blocking the production of these cytokines has improved hemodynamic performance and the sequential organ functions. Thus, in the present work, the amelioration effect of the hepatic tissues by SAV seemed to be mediated by the blocking of the pro-inflammatory cytokines through inhibition of NF-κB.

Fas is a type I membrane receptor belonging to a member of the TNF-receptor superfamily. Activation of Fas by Fas ligand induces apoptosis via activation of the caspase cascade [35]. It was found that Fas engagement with Fas ligand induced activation of Akt and upregulation of endothelial nitric oxide synthase expression without induction of apoptosis [36]. Similarly, here we found that upregulation of Fas induced activation of Akt1 in LPS-only group. The upregulation of AKT1 was markedly elevated by SAV through Fas signaling in LPS + 100 SAV group as well. Restoration behavior of SAV was also confirmed in the terms of the partially and completely restoring of TNF-α and IFN-γ mRNA expression to the normal levels, respectively. An Akt1-dependent pathway contributes to the full activation of IFN-stimulated genes by relieving their repression by EMSY and BRCA2 [37]. Similar data established that Akt activity is essential for upregulation of key IFN-α- and IFN-γ-inducible proteins, which have important functional consequences in the induction of IFN responses [38]. In addition, retrovirus-mediated expression of activated Akt in primary T cells from CD28-deficient mice is capable of selectively restoring production of IL-2 and IFN-γ [39].

Akt is critical for cell survival triggered by growth factors, extracellular matrix, and other stimuli [40]. It is proved in this work that activation and upregulation of AKT1 was accompanied with a significant decrease of lymphocyte percentage in the peripheral blood. Proper regulation of T cell death is of vital importance for the function of the immune system. The death of activated peripheral T cells is crucial for processes such as down-modulation of immune responses after clearance of infectious agents (LPS), peripheral tolerance, and maintenance of immune-privileged sites. These processes are largely proceeding due to the enhanced susceptibility of activated T cells to spontaneous, activation-, and Fas-induced apoptosis [41].

Upregulation of AKT1 by SAV was accompanied with improvement of liver histological architecture and functions, blood level of lipids and oxidative stability. SAV significantly decreased the elevated levels of cholesterol, triglycerides and the inflammatory indicators. A significant increase in arterial elasticity index, a significant improvement in glucose and lipid metabolism, and a significant increase in HDL-cholesterol were also observed in patients who treated with antioxidants [42]. Additionally, the beneficial effect of antioxidant supplementation on LDL oxidation has been demonstrated [43]. This was clear in the improvement of the hepatic tissues histological structure restoration induced by the 100 μg/kg dose of SAV, which successfully ameliorated the inflammatory action of LPS. On contrary, increasing the dose of SAV to 600 μg/kg synergistically with LPS, had negatively impacted the rats, both physiologically and histo-pathologically, that resulted in the high mortality rate recorded in this group. This suggestion was logically accepted when we found that the dose of 600 μg/kg without LPS caused a high oxidative stress with clear hepatic tissue damages.

In conclusion, this study proved anti-oxidant and anti-inflammatory bioactivities of SAV at the dose of 100 μg/kg body weight. Therefore, SAV can be considered as having a new property of enhancing immunogenicity in vivo. Data suggests that SAV is capable of alleviating LPS-induced oxidative stress and tissue damage. Although, significant reductions in the number of the inflammatory cellular infiltration in liver were proved in this study, overcoming the detailed regulatory mechanisms underlying these events are needed. It is proved in this study that Fas signaling regulates generation and activation of the survival signal, Akt1. Thus, Fas may be a therapeutic target for SAV in inflammatory pathological conditions. The chemical constituents and the mechanisms responsible for the pharmacological activities are still yet to be investigated. The approach that we have described currently needs to be more investigated, to help finding and confirming the detailed bioactive effects of the SAV.

## Abbreviations

AKT1, Protein kinase B; AOPP, Advanced oxidation protein products; IFN-γ, Interferon gamma; IL-1, Interleukin-1; GGT, Gamma-glutamyltranspeptidase; LPS, Lipopolysaccharides; MAD, Lipid peroxidation (Malondialdehyde); SAV, Samsum ant venom; TNF-α, Tumor necrosis factor-1α.

## Competing interests

The authors declare no conflicts of interest.

## Authors’ contributions

HE designed the study, described result changes, prepared figures, drafted the manuscript and performed the statistical analysis. MA was responsible for fund support via project no. NLCP-1/2009. AI was responsible for English writing editing. SG was responsible for animal care and groups. All authors read and approved the final manuscript.
